# Single-cell transcriptomic analysis identifies vascular smooth muscle cell transcriptional programs associated with human carotid plaque remodeling

**DOI:** 10.3389/fmolb.2026.1950061

**Published:** 2026-09-25

**Authors:** Yi-Jie Lu, Xue-Guang Lin, Ting Wang, Fan-Xuan Huang, Ying Deng, Shuai Jiang, Jing Dong Tang, Bo Yu

**Affiliations:** 1 Department of Vascular Surgery, Shanghai Key Laboratory of Vascular Lesions Regulation and Remodeling, Fudan University Pudong Medical Center, Shanghai, China; 2 Fudan Zhangjiang Institute, Shanghai, China; 3 Shanghai Fourth People’s Hospital, School of Medicine, Tongji University, Shanghai, China; 4 Shanghai General Hospital, Shanghai Jiao Tong University School of Medicine, Shanghai, China

**Keywords:** atherosclerosis, carotid plaque, plaque rupture, regulon activity, single-cell RNA sequencing (scRNA-seq), transcriptional continuum, transcriptional program, vascular smooth muscle cell (VSMC)

## Abstract

**Background:**

Vascular smooth muscle cells (VSMCs) contribute to plaque structure and exhibit diverse transcriptional programs in human atherosclerosis. Although human plaque single-cell studies have established substantial VSMC heterogeneity, an important unresolved question is whether discovery-defined programs remain recognizable across independent patients and sequencing platforms without forced integration, and whether those programs retain associations with plaque instability. We therefore focused on program-level reproducibility, inferred regulatory activity, continuous expression organization, and rupture-associated variation.

**Methods:**

We used three public human carotid plaque transcriptomic datasets in nonredundant analytical roles rather than merging all single-cell cohorts into a single atlas. GSE253903, a 10x Genomics single-cell cohort, was used for broad plaque mapping and focused VSMC characterization. SCENIC and transcriptional-continuum analyses were restricted to finalized VSMCs. Discovery signatures and assignment rules were frozen before independent assessment in the Smart-seq2 dataset GSE260657, with uncertain cells permitted to remain unassigned. GSE120521 supported exploratory paired comparison between relatively stable and visibly ruptured plaque regions.

**Results:**

Quality control retained 46,209 cells from 12 patients in GSE253903, including 3,784 primary VSMCs after removal of immune-like contaminants. Five descriptive VSMC programs were identified: canonical contractile, extracellular-matrix-associated/fibromyocyte-like, DLX-osteomodulated, stress-responsive, and interferon-associated. VSMC-restricted SCENIC analysis showed higher inferred *ATF3, JUND,* and *CREB5* regulon activity in the stress-responsive program and higher *TEAD1, KLF2,* and *XBP1* activity in the DLX-osteomodulated program. Pseudotime analysis described two transcriptional continua with shared canonical contractile, stress-responsive, and extracellular-matrix-associated organization. In GSE260657, 842 of 1,483 VSMC-enriched cells were confidently projected, while 641 remained unassigned. Six of seven symptomatic-versus-asymptomatic program effects had concordant directions across the single-cell datasets, although no GSE260657 module reached a false-discovery rate below 0.05. In four paired plaques, ruptured regions showed lower contractile scores and higher osteogenic, foam-cell-like, and proliferation scores. qPCR analysis of an independent paired plaque cohort provided orthogonal tissue-level RNA support for selected remodeling-associated gene patterns.

**Conclusion:**

Human carotid plaque VSMCs display heterogeneous transcriptional programs with program-associated inferred regulon activity and continuous expression organization. An uncertainty-aware, cross-platform assessment supported partial conservation of these programs and linked selected remodeling patterns to plaque instability. This framework is intended to complement, rather than replace, spatial and mechanistic studies by identifying reproducible program-level features that can be prioritized for localization and functional testing.

## 1 Introduction

Carotid atherosclerosis is a major cause of ischemic cerebrovascular disease. Plaque behavior is not determined by luminal narrowing alone: lesions of similar anatomical severity can differ in cellular composition, extracellular-matrix organization, inflammatory activity, and susceptibility to disruption (Libby, 2021; Bentzon et al., 2014; Finn et al., 2010). Rupture exposes thrombogenic material and can precipitate embolic events, making the molecular heterogeneity of human carotid lesions clinically relevant (Bentzon et al., 2014; Finn et al., 2010; Virmani et al., 2000; Redgrav et al., 2006). Transcriptomic profiling offers an opportunity to examine this heterogeneity directly in human tissue and to identify cellular programs associated with plaque remodeling and instability.

Vascular smooth muscle cells (VSMCs) are central to plaque architecture and fibrous-cap integrity (Bennett et al., 2016; Basatemur et al., 2019; Grootaert and Bennett, 2021; Owens et al., 2004). In the healthy arterial wall, VSMCs maintain a contractile phenotype characterized by smooth-muscle structural and regulatory genes. During atherosclerosis, VSMCs can undergo phenotypic remodeling accompanied by reduced contractile features and increased extracellular-matrix (ECM), inflammatory, lipid-associated, stress-response, or osteogenic gene expression (Bennett et al., 2016; Basatemur et al., 2019; Grootaert and Bennett, 2021; Owens et al., 2004; Shankman et al., 2015; Cherepanova et al., 2016; Alencar et al., 2020; Wirka et al., 2019). Lineage-tracing and functional studies have shown that modulated VSMCs can adopt macrophage-like, fibromyocyte-like, and osteochondrogenic features, while retaining context-dependent protective or adverse roles (Shankman et al., 2015; Cherepanova et al., 2016; Alencar et al., 2020; Wirka et al., 2019; Feil et al., 2014; Allahverdian et al., 2014; Vengrenyuk et al., 2015; Durham et al., 2018; Speer et al., 2009). Human single-cell studies likewise demonstrate substantial diversity within the plaque VSMC compartment (Fernandez et al., 2019; Depuydt et al., 2020; Pan et al., 2020; Slenders et al., 2022; Bashore et al., 2024; Mocci et al., 2024), but observed expression patterns may form gradients rather than sharply separated populations. A transcriptional-program framework therefore provides a cautious way to describe VSMC heterogeneity without assuming that each pattern represents a distinct biological subtype.

Recent human plaque studies have substantially expanded the cellular catalogue of atherosclerosis through multimodal single-cell profiling, gene-regulatory-network analysis, and, more recently, single-cell spatial transcriptomics (Bashore et al., 2024; Mocci et al., 2024; Pauli et al., 2025). Spatial methods add essential information about where cells and transcriptional states are located within plaque microenvironments, but they do not by themselves resolve whether a program defined in one cohort is reproducible in another cohort, whether its apparent boundaries are platform dependent, or how uncertainty should be handled during transfer. Individual plaque cohorts remain modest in size, and differences in clinical composition, tissue processing, cell selection, sequencing chemistry, and transcript coverage can influence detected programs (Fernandez et al., 2019; Depuydt et al., 2020; Pan et al., 2020; Slenders et al., 2022; Bashore et al., 2024; Mocci et al., 2024; Pauli et al., 2025). Droplet-based 10x Genomics and plate-based Smart-seq2 data differ substantially in sensitivity and scale. Simply pooling public datasets can increase cell numbers but may blur platform-specific structure and encourage retrospective relabeling. We therefore treated reproducibility as a distinct analytical question: define and freeze a program framework in one cohort, project it into an independent platform, and allow low-confidence cells to remain unassigned rather than forcing every cell into a discovery label. Regulatory-network and transcriptional-continuum approaches provide complementary views of VSMC organization that are distinct from spatial localization. SCENIC estimates transcription factor-associated regulon activity from co-expression and motif information (Aibar et al., 2017; Huynh-Thu et al., 2010; Van de Sande et al., 2020), allowing comparison of inferred regulatory activity across transcriptional programs; these estimates identify candidate regulatory associations rather than direct regulation. Slingshot and related trajectory methods organize cells along fitted expression geometry and can reveal continuous changes in module scores and genes (Street et al., 2018; Van den Berge et al., 2020). In cross-sectional human plaques, however, pseudotime is not biological time and should be interpreted as a transcriptional continuum rather than a lineage or developmental sequence. Together, these analyses can prioritize reproducible regulatory and expression features for subsequent spatial and perturbational validation. Accordingly, the aim of this study was not to construct another pooled plaque atlas, but to test whether a discovery-defined VSMC transcriptional-program framework could be characterized at the regulatory and continuous-expression levels, transferred across an independent sequencing platform while preserving assignment uncertainty, and related to clinically relevant plaque instability. GSE253903 served as the single-cell discovery cohort (Bashore et al., 2024), GSE260657 provided independent Smart-seq2 assessment of frozen discovery signatures (Mocci et al., 2024), and GSE120521 enabled exploratory within-patient analysis of rupture-region associations (Mahmoud et al., 2019). Targeted qPCR in an independent paired plaque cohort provided orthogonal RNA-level support for selected remodeling-associated genes.

## 2 Methods

### 2.1 Public datasets and study design

Candidate public datasets were evaluated for a prespecified analytical role rather than pooled solely to maximize cell number. Eligibility required human carotid atherosclerotic tissue, publicly accessible expression matrices, resolvable patient/sample provenance, sufficient representation of the VSMC compartment for the intended analysis, and a design that contributed a nonredundant role to discovery, cross-platform assessment, or plaque-instability analysis. Datasets were not merged into the discovery cohort when doing so would compromise the separation between discovery and validation, duplicate overlapping source material, or introduce a fundamentally different biological contrast that could not be interpreted on the same scale. Three datasets deposited in the NCBI Gene Expression Omnibus (GEO) met the role-specific criteria used in the primary analysis (Clough et al., 2024). GSE253903 comprised 10x Genomics scRNA-seq data from carotid plaques of 12 patients and served as the discovery cohort (Bashore et al., 2024). GSE260657 comprised Smart-seq2 gene-by-cell matrices from 15 carotid endarterectomy specimens and provided an independent sequencing platform for frozen-signature assessment (Mocci et al., 2024). GSE120521 comprised bulk RNA sequencing from paired relatively stable and visibly ruptured plaque regions of four patients and supported exploratory rupture-associated analysis (Mahmoud et al., 2019). GSE125771 was not eligible for single-cell program definition because it is a bulk microarray dataset. We also re-reviewed additional public human carotid single-cell resources during revision. GSE159677 contains paired calcified atherosclerotic-core and proximal-adjacent carotid tissue from three patients (Alsaigh et al., 2022); its small paired cohort and plaque-versus-adjacent-tissue contrast represent a different biological comparison from the symptom-based and rupture-associated analyses used here. The Maegdefessel/Reilly-associated resources GSE247238 and GSE294291 provide additional human carotid scRNA-seq cohorts, and GSE294466 provides Xenium spatial transcriptomics; these resources are explicitly acknowledged through the recent integrated human carotid study (Pauli et al., 2025). They were not pooled into the present discovery cohort because the primary design intentionally separated a 10x discovery cohort from an independent Smart-seq2 transfer cohort, and spatial localization was outside the prespecified primary analysis. The inclusion/exclusion decision was therefore based on analytical role and comparability rather than on an assumption that all public single-cell datasets should be merged.

Race/ethnicity metadata were reviewed where publicly available. In the GEO records for GSE253903, nine of 12 samples are annotated as Caucasian (spelled “Caucacian” in the source records) and three as unknown; race/ethnicity is not reported in the public GSE260657 sample records. Race/ethnicity was not treated as a sequencing-technical covariate because it does not operate like library chemistry, read depth, or tissue-processing batch. Nevertheless, genetic ancestry and correlated environmental, clinical, and treatment exposures may contribute to biological heterogeneity across cohorts. Because the available metadata are incomplete and the discovery cohort contains little reported racial diversity, ancestry- or race-stratified analyses would be underpowered and potentially misleading. We therefore did not impute missing ancestry and explicitly recognize limited population diversity as a constraint on generalizability.

### 2.2 Single-cell preprocessing and VSMC identification

GSE253903 Cell Ranger matrices were processed separately by sample. Quality control considered detected features, unique molecular identifier counts, mitochondrial, ribosomal, and hemoglobin transcript fractions, and library complexity. Sample-specific thresholds combined distribution-based bounds with predefined safety limits; doublets were identified within samples using scDblFinder (Germain et al., 2021). Retained cells were log normalized, scaled using variable genes, and analyzed by principal-component analysis, sample-aware Harmony correction (Korsunsky et al., 2019), nearest-neighbor graph construction, UMAP (McInnes et al., 2018), and graph-based clustering. Detailed thresholds and parameters are provided in Supplementary Methods.

Broad lineages were annotated by jointly reviewing predefined marker-set scores and cluster-level positive markers. The framework included vascular, stromal, lymphoid, myeloid, dendritic, mast, neutrophil, and cycling compartments, with VSMCs distinguished from pericytes and fibroblasts. Broad-atlas VSMC clusters were extracted and reprocessed; a 29-cell cluster expressing *PTPRC*, *TYROBP*, *C1QC*, and *SPI1* was excluded as myeloid contamination. In GSE260657, VSMC-enriched cells were required to detect at least three of *MYH11*, *CNN1*, *TAGLN*, *ACTA2*, *MYL9*, and *SMTN*, including *MYH11* or *CNN1*, and to fall within the upper within-patient quartile of the VSMC identity score. Immune, endothelial, pericyte, and fibroblast marker programs were used to exclude obvious contamination. A contaminating program had to be jointly detected and substantially exceed the VSMC identity score; isolated ECM-gene expression was insufficient for exclusion because ECM-associated remodeling was under assessment.

### 2.3 VSMC program identification and module scoring

GSE253903 VSMCs were reprocessed independently using log normalization, variable-gene principal-component analysis, Harmony correction by sample, UMAP, and graph-based clustering. Positive markers were identified using the Wilcoxon rank-sum test as implemented in Seurat (Satija et al., 2015; Stuart et al., 2019; Hao et al., 2021). Descriptive labels integrated cluster markers with predefined module scores and comprised canonical contractile, ECM-associated/fibromyocyte-like, DLX-osteomodulated, stress-responsive, and interferon-associated programs. Seven literature-informed modules—contractile, fibromyocyte/ECM, inflammatory, osteogenic, foam-cell-like, proliferation, and stress response—were defined and frozen before assessment of GSE260657 and GSE120521. Scores were summarized at cluster and patient levels; program definitions and module genes are provided in the supplementary tables.

### 2.4 SCENIC and transcriptional continuum analysis

SCENIC was restricted to the 3,784 finalized GSE253903 VSMCs; whole-plaque populations were not analyzed. Network inference used cells sampled proportionally within sample-by-program strata, GENIE3 (Huynh-Thu et al., 2010), motif-based pruning, and AUCell activity scoring (Aibar et al., 2017). Activity was calculated for all finalized VSMCs, summarized within sample-program combinations, and analyzed using limma (Ritchie et al., 2015) with program and sample terms. Each program was contrasted with the mean of the remaining programs, with false-discovery-rate control within each contrast. This sample-adjusted framework avoided treating individual cells as independent clinical replicates. Database provenance, implementation parameters, sampled-cell and input-gene records, and the complete audit trail are reported in Supplementary Methods. Results represent program-associated inferred regulon activity, not direct binding or causal regulation.

Slingshot (Street et al., 2018) was applied to the finalized VSMC UMAP and descriptive program labels, with the canonical contractile program specified as the root because it showed the strongest canonical smooth-muscle expression; no terminal state was imposed. Two fitted curves were inferred. Gene-level changes along the curves were modeled using tradeSeq negative-binomial generalized additive models (Van den Berge et al., 2020), and predefined module trends were evaluated using generalized additive models and weighted-cell Spearman correlations. Expressed variable and module genes were included in gene-level modeling. Model parameters, curve-specific values, and weights are provided in Supplementary Methods. Pseudotime was interpreted as continuous expression organization in cross-sectional data, not biological time, lineage progression, or differentiation.

### 2.5 Cross-platform assessment in GSE260657

Projection signatures were derived from the completed GSE253903 analysis and frozen before evaluation of GSE260657. They combined the leading pre-existing cluster markers with predefined module genes, while lower-ranked markers were held out for descriptive assessment. Fixed projection genes were standardized independently within each platform. Each GSE260657 VSMC-enriched cell was compared with the five GSE253903 reference centroids using Pearson similarity. Assignment required both an adequate maximum similarity and sufficient separation from the second-highest similarity; cutoffs were calibrated exclusively in GSE253903. Failure of either criterion produced an explicit unassigned (abstention) outcome rather than a forced label. Thus, unassigned cells were retained as a measure of transfer uncertainty and were not treated as missing data or excluded because they lacked a preferred biological identity. Cross-platform assessment considered assignment yield and confidence, frozen signature-score patterns, centroid correlations, held-out marker direction, and patient-level symptomatic-versus-asymptomatic module-effect direction. Absolute score magnitudes were not assumed to be interchangeable across platforms. Centroid correlations were interpreted descriptively because projected groups were defined using reference-centroid similarity. Full signature construction and confidence cutoffs are provided in Supplementary Methods.

### 2.6 Paired rupture-region analysis

The eight verified GSE120521 columns represented four patient-matched stable and visibly ruptured plaque-region pairs. Duplicate gene symbols were collapsed by mean fragments per kilobase of transcript per million mapped reads, followed by log2(FPKM +1) transformation. Within each frozen module, genes were standardized across the eight samples and averaged to obtain a tissue-level score. Ruptured-minus-stable differences were calculated within patients. Because measurements represented whole-plaque regions, scores were interpreted as tissue-level projections of VSMC-associated programs rather than purified-VSMC expression.

### 2.7 Quantitative PCR analysis of independent plaque samples

Independent paired carotid plaque specimens were obtained from seven patients undergoing carotid endarterectomy. For each patient, tissue samples were collected from relatively stable plaque regions and rupture-associated plaque regions according to pathological assessment. Paired samples from the same patient were analyzed to minimize inter-individual variation. Total RNA was extracted from plaque tissues, reverse-transcribed into cDNA, and analyzed by quantitative real-time PCR using gene-specific primers. *GAPDH* was used as the reference gene. Relative expression levels were calculated using the comparative Ct method (2^-ΔΔCt), with patient-matched stable regions used as calibrators.

### 2.8 Statistical analysis

Patients or paired plaque regions were the units of clinical inference; cells were not treated as independent clinical replicates. Symptomatic-versus-asymptomatic comparisons used patient-level mean differences, 95% confidence intervals, and two-sided Welch t tests separately in GSE253903 and GSE260657. GSE120521 comparisons used paired t tests and Wilcoxon signed-rank tests, with mean and median paired differences, 95% confidence intervals, standardized paired effects, and direction counts. Benjamini–Hochberg correction (Benjamini and Hochberg, 1995) was applied within each cohort or prespecified test family. SCENIC multiplicity correction was performed within each program-versus-rest regulon contrast, and pseudotime gene- and module-level P values were adjusted where applicable. All tests were two sided, and the four-patient rupture-region analysis was interpreted as exploratory.

## 3 Results

### 3.1 A quality-controlled single-cell atlas defines the human carotid plaque VSMC compartment

The study integrated a 10x Genomics scRNA-seq discovery cohort (Bashore et al., 2024), an independent Smart-seq2 cohort (Mocci et al., 2024), and a paired bulk RNA-seq cohort (Mahmoud et al., 2019) (Figure 1A); complete dataset and sample information is provided in Supplementary Table S1. In GSE253903, sample-aware quality control retained 46,209 cells from all 12 patients; detailed sample-level QC distributions are consolidated in Supplementary Figure S1. The retained cells delineated a broad atlas of immune, vascular, and stromal plaque populations (Figure 1B). Canonical markers supported annotation of VSMCs, endothelial cells, macrophage/myeloid cells, T cells, B/plasma cells, fibroblasts, and additional broad classes (Figure 1C). VSMCs were separated from pericytes and fibroblasts before focused extraction. Patient-level cell-class proportions varied substantially (Figure 1D), demonstrating inter-individual plaque heterogeneity and providing an audited framework for focused VSMC analysis.

**FIGURE 1:**
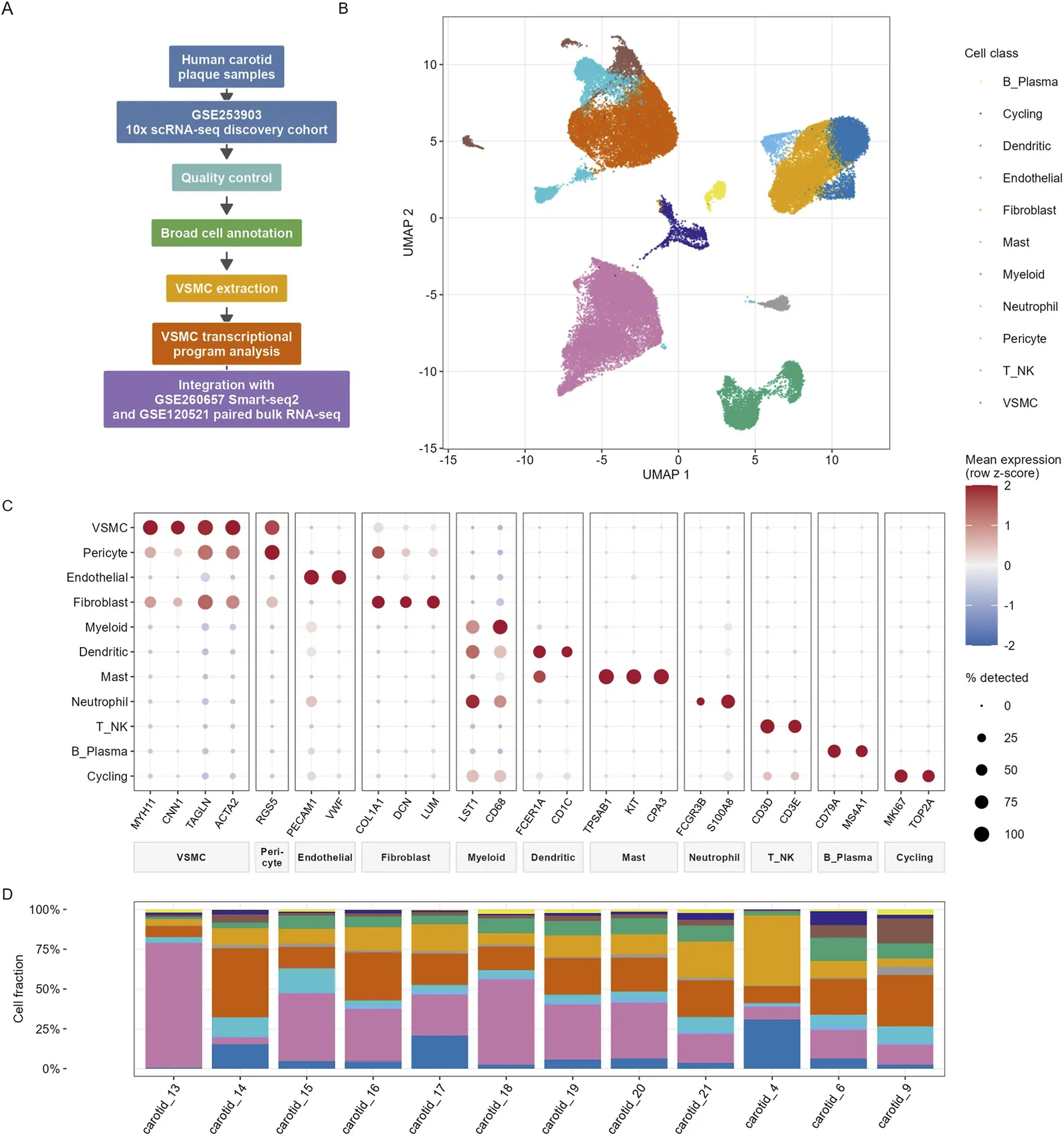
Single-cell atlas of human carotid plaque. **(A)** Study workflow showing the GSE253903 10x Genomics scRNA-seq discovery cohort, sample-aware quality control, broad cell annotation, VSMC extraction and transcriptional-program analysis, followed by cross-platform assessment in GSE260657 Smart-seq2 data and paired analysis of GSE120521 bulk RNA-seq plaque regions. **(B)** UMAP of 46,209 QC-retained GSE253903 plaque cells colored by broad annotated cell class. **(C)** Dot plot of representative broad-lineage markers. Markers are ordered by lineage, with canonical VSMC markers grouped together; mast-cell identity is represented by mast-cell-associated markers in the revised panel. Dot size represents the percentage of cells with detected expression; color represents mean expression standardized within each gene. **(D)** Patient-level proportions of broad cell classes across 12 GSE253903 patients. Colors denote broad cell classes. Proportions are descriptive, and no patient-level abundance test was performed. Detailed sample-level QC metrics that were previously shown in the main figure are consolidated in Supplementary Figure S1.

### 3.2 Focused analysis identifies five VSMC transcriptional programs

Broad-atlas annotation identified 3,813 VSMC candidates across all 12 patients. Focused reclustering detected a 29-cell cluster expressing leukocyte and myeloid markers, which was excluded as contamination. The finalized object therefore contained 3,784 primary VSMCs (Figures 2A,B).

**FIGURE 2:**
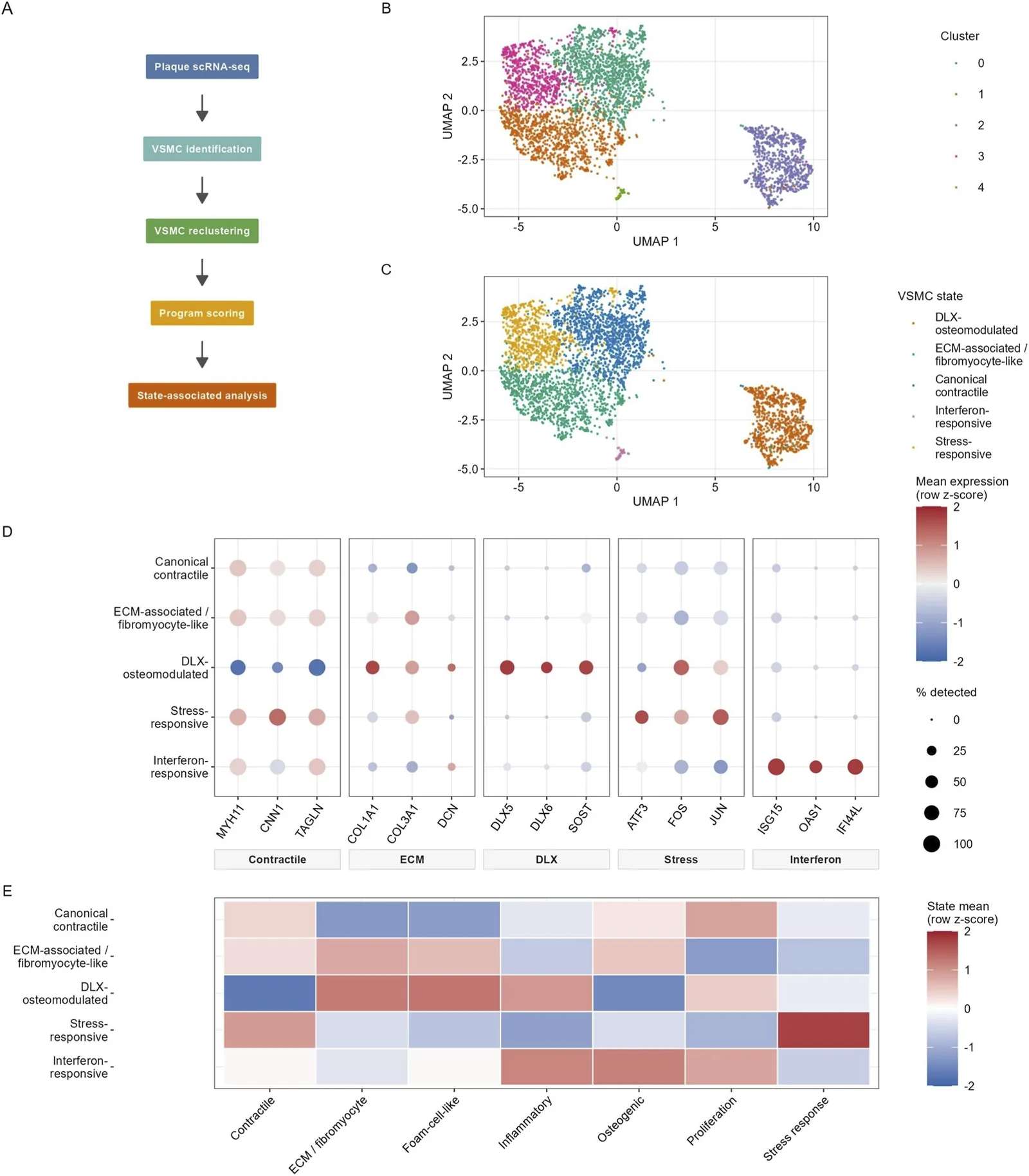
VSMC transcriptional programs in human carotid plaques. **(A)** Workflow for broad-atlas VSMC extraction, focused reclustering, marker assessment, program scoring, and program-associated analyses. **(B)** UMAP of 3,784 primary GSE253903 VSMCs colored by unsupervised cluster. **(C)** UMAP colored by five descriptive VSMC transcriptional programs: canonical contractile, ECM-associated/fibromyocyte-like, DLX-osteomodulated, stress-responsive, and interferon-associated. The same color mapping is used for programs throughout figure. **(D)** Dot plot of representative program-associated markers, ordered in biologically coherent blocks corresponding to the five programs. Dot size represents the percentage of cells with detected expression; color represents row-standardized mean expression. **(E)** Heatmap of mean predefined module scores across the five VSMC programs. Color represents scores standardized within each module. Patient-level program distributions are presented in Supplementary Figure S2D as a descriptive re-analysis rather than a novel abundance result.

Five descriptive transcriptional programs occupied partially overlapping regions of the VSMC embedding (Figure 2C). The canonical contractile program showed strong smooth-muscle-associated expression and the highest contractile module activity. The ECM-associated/fibromyocyte-like program retained contractile features while displaying increased ECM-associated scores. The DLX-osteomodulated program expressed *DLX5*, *DLX6*, *SOST*, and *PTN* and combined lower contractile features with elevated ECM-associated expression. The stress-responsive program expressed genes including *FSTL3*, *HSPA1B*, and *TNFRSF12A*. A small interferon-associated program expressed *OAS1*, *IFI44L*, *ISG15*, and related interferon-response genes. Marker expression and module-score patterns supported these annotations (Figures 2D,E).

The major VSMC programs were detected across multiple patients, although their relative distributions varied. To avoid presenting a descriptive re-analysis of patient-level program composition as a new abundance result, the patient-level distribution is shown in Supplementary Figure S2D rather than the main figure. The interferon-associated program was rare, comprising 41 cells across 11 samples. Collectively, these findings demonstrate heterogeneous yet overlapping VSMC transcriptional organization in human carotid plaques. The program labels summarize expression patterns and do not denote novel cell subtypes.

### 3.3 VSMC programs show program-associated inferred regulon activity

SCENIC analysis restricted to the finalized VSMC compartment retained 28 motif-supported regulons (Figures 3A,B). Inferred regulon activities differed among the five programs after accounting for sample effects. The stress-responsive program showed higher inferred activity of *ATF3, JUND,* and *CREB5* regulons (Figure 3C). In contrast, the DLX-osteomodulated program showed higher *TEAD1, KLF2,* and *XBP1* activity. *TEAD1* activity was also higher in the ECM-associated/fibromyocyte-like program.

**FIGURE 3:**
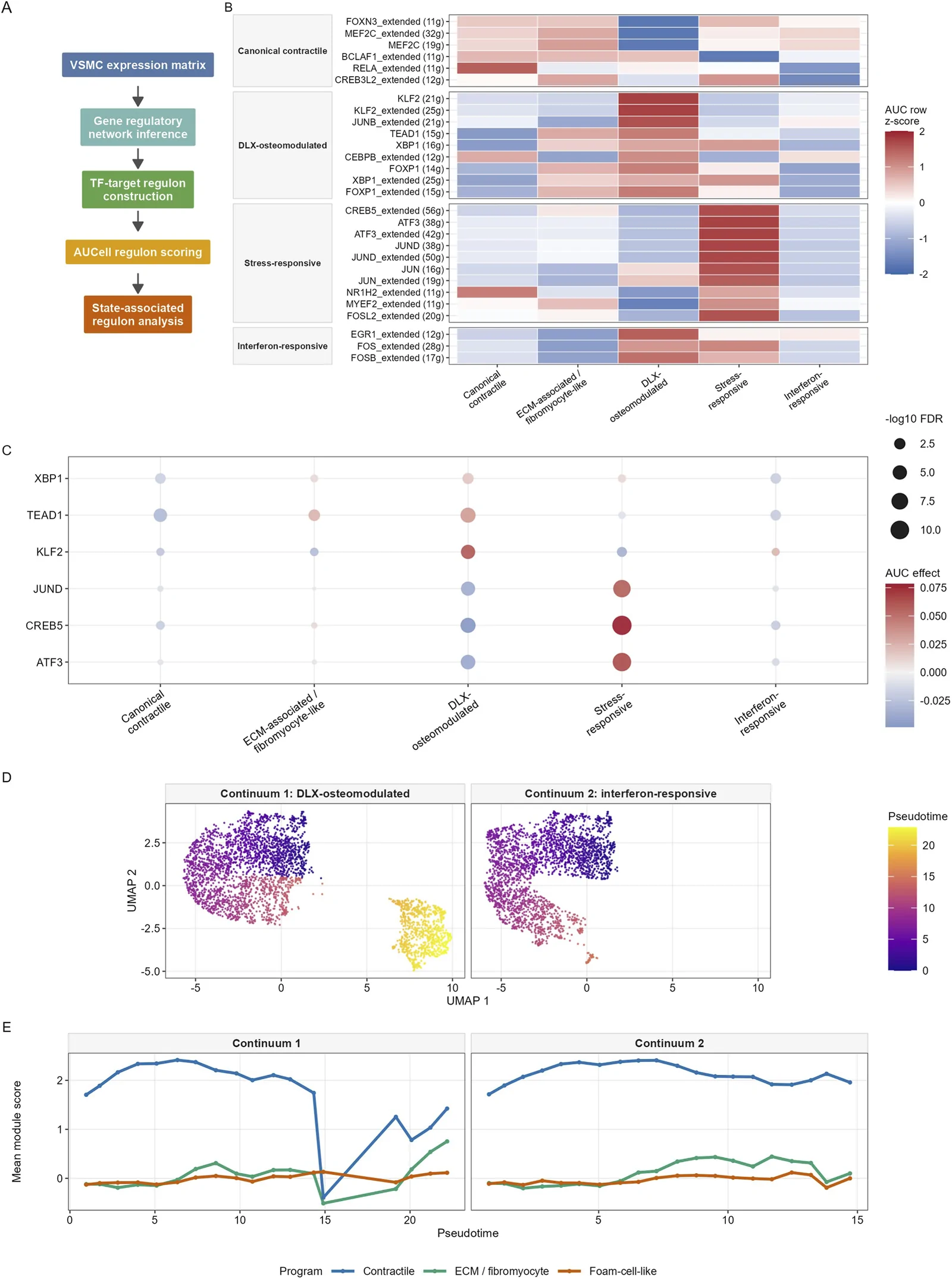
Regulatory landscape and transcriptional continua of VSMC remodeling. **(A)** SCENIC workflow restricted to the finalized GSE253903 VSMC subset, including network inference, motif pruning, AUCell scoring, and sample-adjusted program comparisons. **(B)** Heatmap of motif-supported regulon activity across the five VSMC programs, with regulons ordered by their dominant program-associated activity to improve visual interpretation; the complete 28-regulon presentation and statistics remain available in Supplementary Figure S3; Supplementary Table S2. Color represents activity standardized within each regulon. **(C)** Program-versus-rest inferred activity estimates for *ATF3, JUND, CREB5, TEAD1, KLF2,* and *XBP1* regulons. Color represents the activity estimate and point size represents -log10 of the Benjamini-Hochberg-adjusted P value. **(D)** Slingshot pseudotime projected onto the VSMC UMAP for the DLX-associated and interferon-associated transcriptional continua. Color represents relative pseudotime within each fitted curve. **(E)** Binned mean contractile, ECM/fibromyocyte, and foam-cell-like module scores along each continuum. Lines show the binned mean trends; colors identify modules. These panels describe program-associated inferred regulon activity and transcriptional continua, not direct regulation, biological time, or a developmental sequence.

These patterns link the VSMC transcriptional programs to distinct candidate regulatory associations. The complete program-versus-rest statistics and false-discovery rates are provided in Supplementary Table S2. Because SCENIC integrates co-expression and motif information, the results represent program-associated inferred regulon activity rather than direct evidence that these transcription factors control plaque VSMC remodeling.

### 3.4 VSMC programs are organized along two transcriptional continua

Slingshot fitted two continua within the existing VSMC expression geometry (Figure 3D). Both shared an organization extending from the canonical contractile region through stress-responsive and ECM-associated regions. One continuum extended toward the DLX-osteomodulated program, whereas the second extended toward the interferon-associated program. This shared structure supported gradual transcriptional organization across programs rather than fully isolated expression patterns.

Along the DLX-associated continuum, the contractile module decreased while ECM/fibromyocyte and foam-cell-like modules increased (Figure 3E). The interferon-associated continuum also showed increasing ECM/fibromyocyte and foam-cell-like scores, whereas its contractile score did not show a corrected monotonic association. Notably, the predefined osteogenic module did not increase monotonically along either curve despite the DLX-osteomodulated endpoint label, indicating that the program cannot be reduced to a single linear osteogenic process.

Multiple contractile, ECM, stress, and interferon-related genes varied along the fitted curves; complete gene-level results are reported in Supplementary Table S3. These analyses describe transcriptional continua in cross-sectional expression space and do not establish temporal progression or developmental relationships.

### 3.5 Independent Smart-seq2 data show partial cross-platform conservation

GSE253903 program signatures and projection thresholds were frozen before independent assessment in GSE260657 (Figure 4A). Marker-based selection and contamination exclusion retained 1,483 VSMC-enriched Smart-seq2 cells across 15 patients. Each cell was compared with the five discovery centroids, and ambiguous cells were not forced into an assignment.

**FIGURE 4:**
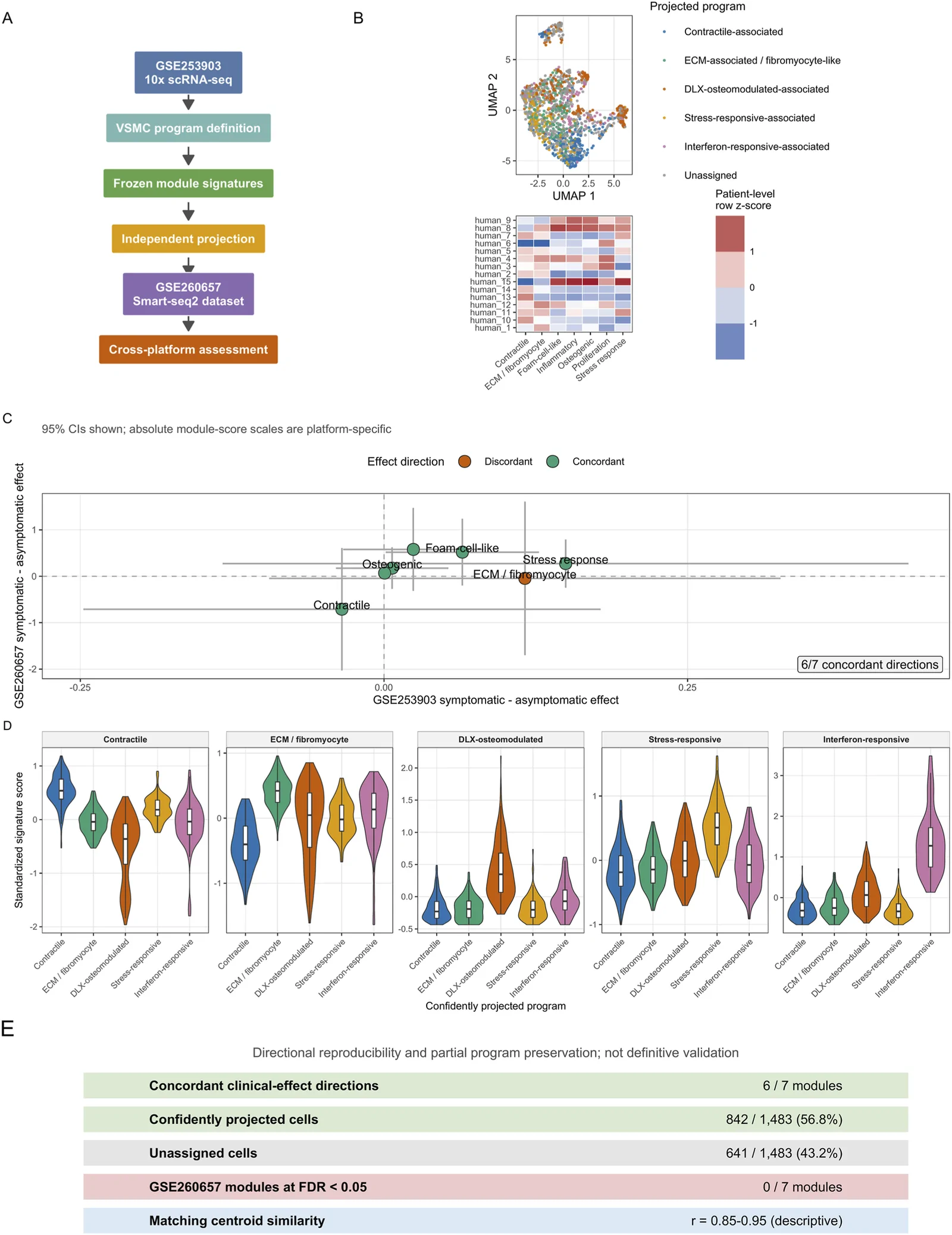
Cross-platform conservation of human carotid plaque VSMC transcriptional programs. **(A)** Workflow showing definition and freezing of GSE253903-derived VSMC program signatures before independent projection to GSE260657 Smart-seq2 data. **(B)** GSE260657 VSMC-enriched-cell UMAP colored by confidently projected program, with ambiguous cells shown as unassigned (top), and patient-level predefined-module score landscape (bottom). Program colors match Figure 2; heatmap color represents the within-panel module-score scale. **(C)** Symptomatic-minus-asymptomatic module associations in GSE253903 and GSE260657. Points represent patient-level association estimates and horizontal error bars represent 95% confidence intervals. Colors identify datasets; the annotation indicates that six of seven modules had concordant directions. Magnitudes are expressed on platform-specific standardized scales. **(D)** Violin plots of standardized frozen signature scores across confidently projected GSE260657 programs. Each panel represents one frozen program signature, and the distributions are shown across the five confidently projected VSMC programs. **(E)** Cross-platform assessment summary showing confident and unassigned projection counts, directional concordance, centroid similarity, held-out marker overlap, and false-discovery-rate status. Of 1,483 cells, 842 were confidently projected and 641 remained unassigned; no GSE260657 module reached FDR <0.05. Centroid correlations are descriptive because projected groups were defined using reference-centroid similarity. The figure supports partial cross-platform reproducibility and transcriptional similarity, not definitive validation or identical program identity.

Of the 1,483 cells, 842 (56.8%) were confidently projected and 641 (43.2%) remained unassigned (Figures 4B,E). This unassigned fraction reflects the conservative abstention rule: cells failing either the calibrated maximum-similarity threshold or the top-versus-second-best separation threshold were deliberately left without a discovery-program label. Supplementary Figure S4 shows the distribution of assigned and unassigned cells across the embedding and patients together with the two confidence metrics. Frozen signature-score patterns among assigned cells were broadly consistent with their projected programs (Figure 4D). Reference-to-projected centroid correlations and held-out markers further supported transcriptional similarity, although both were interpreted within the constraints of the projection framework. At the patient level, six of seven symptomatic-minus-asymptomatic program associations had concordant directions between GSE253903 and GSE260657 (Figure 4C). Contractile scores were lower in symptomatic patients in both datasets, whereas foam-cell-like, inflammatory, osteogenic, proliferation, and stress-response scores were higher; the ECM/fibromyocyte association was discordant. No GSE260657 module reached FDR <0.05 (Figure 4E). The independent dataset therefore supported the partial cross-platform reproducibility of predefined VSMC transcriptional programs.

### 3.6 Ruptured plaque regions are associated with altered VSMC program scores

GSE120521 comprised matched stable and visibly ruptured plaque regions from four patients, yielding eight regional samples and four within-patient comparisons (Figure 5A). Frozen VSMC program scores were projected into the bulk RNA-seq data and interpreted at tissue level.

**FIGURE 5:**
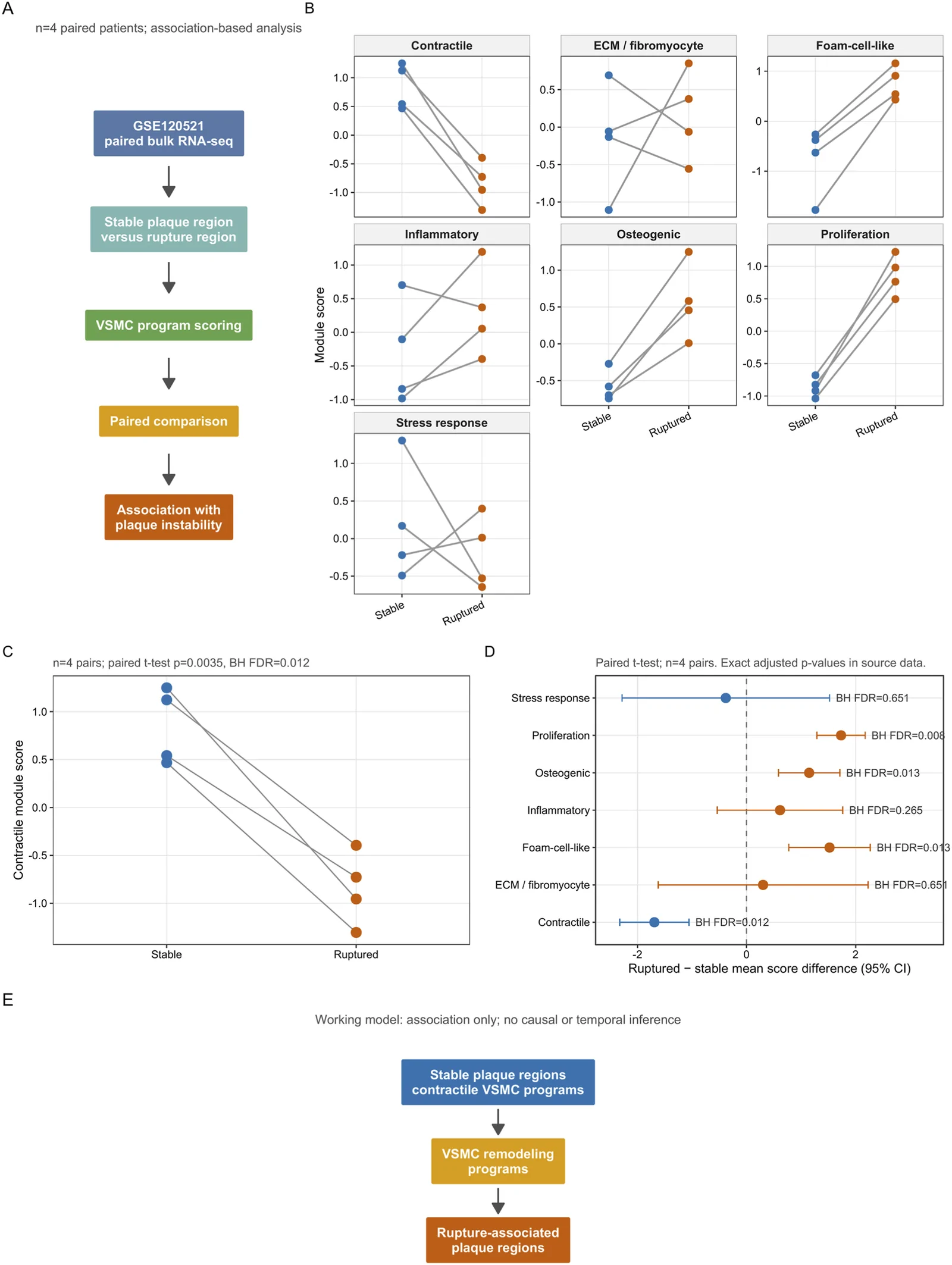
Association of VSMC programs with plaque rupture regions. **(A)** Paired GSE120521 study design comparing stable and visibly ruptured plaque regions from four patients using frozen VSMC program scores. **(B)** Individual paired scores for all seven predefined programs. Lines connect stable and ruptured regions from the same patient; point colors identify plaque region. **(C)** Paired contractile-program scores. All four patients showed lower scores in the ruptured region. Lines connect patient-matched samples. **(D)** Ruptured-minus-stable mean score differences for the seven programs. Points represent mean paired differences and horizontal error bars represent 95% confidence intervals; labels report paired t-test Benjamini-Hochberg-adjusted P values. Values above zero indicate higher scores in ruptured regions. **(E)** Association-only working model summarizing lower contractile and higher osteogenic, foam-cell-like, and proliferation program projections in ruptured plaque regions. The analysis is exploratory and does not imply causality or temporal progression.

Contractile scores were lower in the ruptured region in all four patients (Figures 5B,C). The mean ruptured-minus-stable difference was −1.691 (95% CI, −2.327 to −1.056; paired t-test BH-adjusted P = 0.0121). Foam-cell-like scores were higher in all four ruptured regions (mean difference, 1.521; 95% CI, 0.773–2.269; adjusted P = 0.0131), as were osteogenic scores (mean difference, 1.147; 95% CI, 0.583–1.710; adjusted P = 0.0131) and proliferation scores (mean difference, 1.731; 95% CI, 1.289–2.174; adjusted P = 0.00781; Figure 5D). Fibromyocyte/ECM, inflammatory, and stress-response scores did not show corrected paired differences.

The corresponding Wilcoxon signed-rank tests were not significant after correction, consistent with the limited resolution of nonparametric testing with four pairs. Moreover, the bulk-tissue scores may reflect differences in cellular composition as well as expression within VSMCs. Nevertheless, the consistent paired directions associate rupture regions with lower contractile and higher osteogenic, foam-cell-like, and proliferation program projections (Figure 5E). These findings identify rupture-associated remodeling patterns related to VSMC transcriptional programs.

### 3.7 Independent qPCR assessment supports plaque remodeling-associated transcriptional patterns

To provide an independent tissue-level RNA assessment of the computationally identified plaque-remodeling patterns, we performed qPCR analysis of eight representative program-associated genes in paired stable and ruptured plaque regions from seven independent patients. Relative expression patterns were visualized using row-wise standardized heatmaps based on log2-transformed relative expression values (Figure 6A).

**FIGURE 6:**
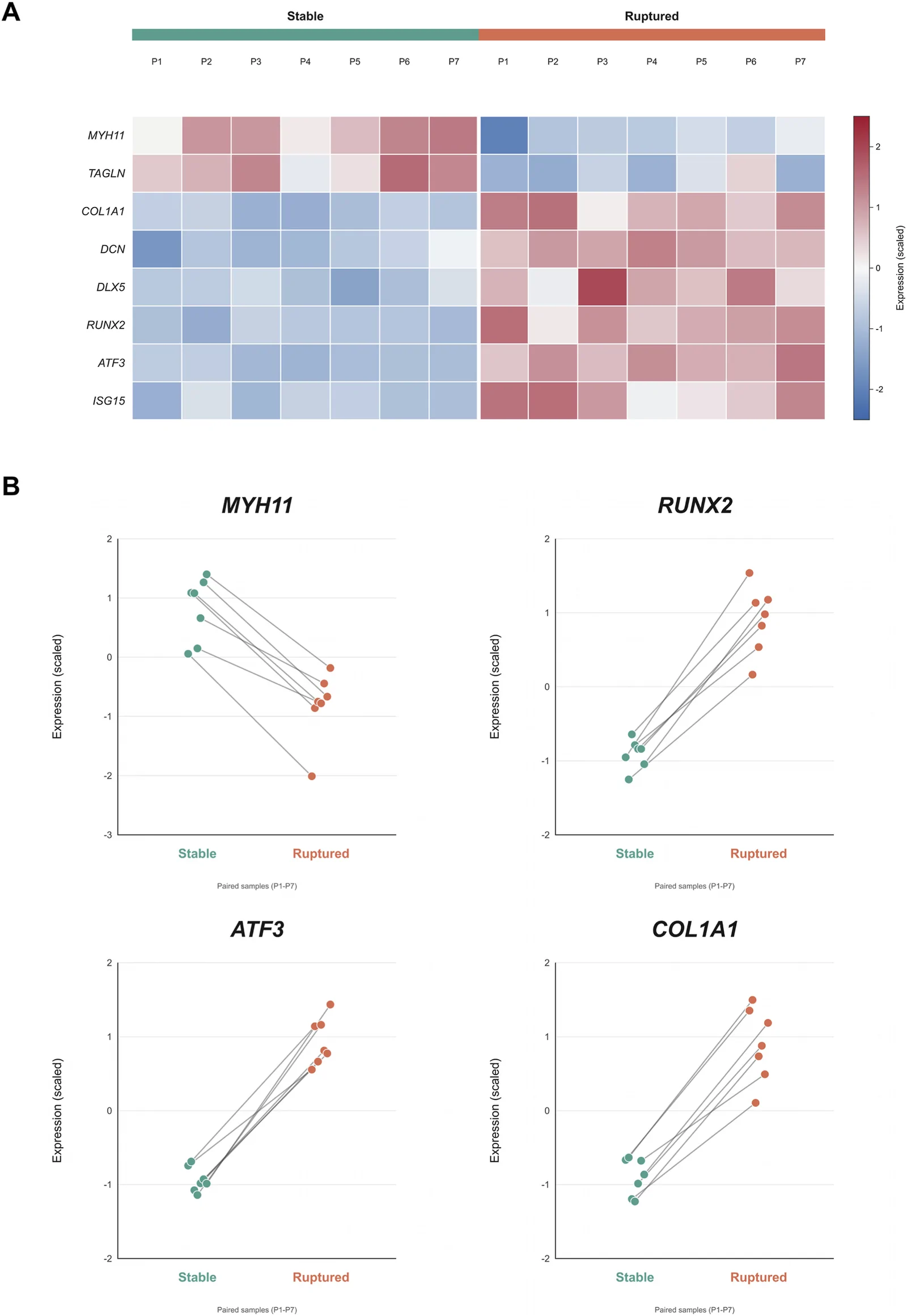
Independent qPCR assessment of plaque remodeling-associated genes. **(A)** Heatmap showing expression patterns of eight selected program-associated genes in paired stable and ruptured plaque regions obtained from seven independent patients undergoing carotid endarterectomy. Relative expression values were calculated using the 2^−ΔΔCT^ method with patient-matched stable regions as calibrators. Values were log2 transformed and standardized by row-wise Z-score normalization using Z=(x−mean)/SD. **(B)** Paired expression plots of representative genes including *MYH11*, *RUNX2*, *ATF3*, and *COL1A1*. Each line connects stable and ruptured regions from the same patient, and points represent individual patient-matched plaque regions. The qPCR analysis provides independent tissue-level support for selected remodeling-associated transcriptional patterns but does not establish VSMC-specific origin because measurements were obtained from whole plaque tissues.

Consistent with the contractile-associated program identified in the single-cell analysis, contractile markers including *MYH11* and *TAGLN* showed reduced expression in ruptured regions across paired samples. In contrast, remodeling-associated genes representing extracellular matrix, osteomodulated, and stress-responsive programs, including *COL1A1*, *DLX5*, *RUNX2*, and *ATF3*, demonstrated increased expression patterns in ruptured regions (Figures 6A,B). Paired expression analyses further showed concordant within-patient directional changes for representative genes associated with contractile loss and plaque remodeling.

The interferon-responsive marker *ISG15* displayed more heterogeneous changes across individual patients, consistent with the context-dependent nature and relatively limited representation of interferon-associated transcriptional programs observed in the single-cell dataset.

## 4 Discussion

This integrative study defines five descriptive VSMC transcriptional programs in human carotid plaques and asks a reproducibility question that is complementary to increasingly detailed single-cell and spatial atlases: which discovery-defined VSMC features remain recognizable across independent patients and sequencing platforms when program definitions are frozen and uncertain cells are not forcibly classified? In the GSE253903 discovery cohort, 3,784 primary VSMCs displayed canonical contractile, ECM-associated/fibromyocyte-like, DLX-osteomodulated, stress-responsive, and interferon-associated expression patterns. VSMC-restricted SCENIC analysis identified program-associated differences in inferred regulon activity, and transcriptional-continuum analysis organized the programs along two partially shared expression geometries. Projection into an independent Smart-seq2 cohort supported partial preservation of the discovery programs while making cell-level uncertainty explicit. Paired analysis of stable and visibly ruptured plaque regions associated rupture with lower contractile-program scores and higher osteogenic, foam-cell-like, and proliferation-program scores. The contribution of the present study therefore lies less in proposing new discrete VSMC cell types than in linking a program-based representation to regulatory associations, continuous expression organization, uncertainty-aware cross-platform transfer, and plaque-instability phenotypes. The five programs capture biologically relevant dimensions of VSMC remodeling. The canonical contractile program reflects preservation of smooth-muscle structural and functional features, whereas the ECM-associated/fibromyocyte-like program combines residual contractile expression with matrix-related genes. This combination may be particularly relevant to plaques because VSMCs contribute extracellular matrix that can influence lesion organization and fibrous-cap structure, while excessive or altered matrix remodeling may also accompany disease progression (Bennett et al., 2016; Basatemur et al., 2019; Grootaert and Bennett, 2021; Owens et al., 2004; Alencar et al., 2020). The DLX-osteomodulated program, characterized by *DLX5*, *DLX6*, *SOST*, and *PTN*, connects reduced contractile expression with matrix- and mineralization-associated features (Alencar et al., 2020; Durham et al., 2018; Speer et al., 2009; Sag et al., 2010). Stress-responsive and interferon-associated programs add further dimensions that may reflect local injury, inflammatory exposure, or cellular adaptation. These patterns align broadly with contractile, matrix-associated, inflammatory, and osteogenic-like VSMC populations described in prior human plaque single-cell studies, including the multimodal atlas of Bashore et al. and the gene-regulatory-network study of (Fernandez et al., 2019; Depuydt et al., 2020; Pan et al., 2020; Slenders et al., 2022; Bashore et al., 2024; Mocci et al., 2024). We therefore do not interpret the five labels as *de novo* cell types. Rather, the program framework deliberately accommodates cells that express more than one biological theme and tests whether such themes can be transferred without redefining them in the validation cohort. A VSMC may retain structural genes while simultaneously increasing matrix, stress, or lipid-associated expression, and these combinations may shift with microenvironmental context. Such overlap is difficult to capture using mutually exclusive labels alone. Patient-level variation may reflect differences in the balance, intensity, or co-occurrence of shared transcriptional processes. The transcriptional-continuum results extend this comparison by showing that canonical contractile, stress-responsive, and ECM-associated expression can occupy shared continuous geometry before extending toward DLX-osteomodulated or interferon-associated patterns. Increasing ECM/fibromyocyte and foam-cell-like scores along both continua suggest that several remodeling features can coexist, while the non-monotonic osteogenic score cautions against reducing the DLX-associated program to a single linear process. The SCENIC analysis provides candidate regulatory associations that complement marker-based annotation. Higher inferred *ATF3, JUND,* and *CREB5* regulon activity in the stress-responsive program is compatible with coordinated immediate-early and cellular-stress transcriptional responses (Hai et al., 2010; Karin, 1995). Higher *TEAD1, KLF2,* and *XBP1* activity in the DLX-osteomodulated program suggests associations involving mechanosensitive transcription, vascular response programs, and proteostatic signaling (Liu et al., 2014; Atkin et al., 2007; Hetz et al., 2020). *TEAD1* was also associated with the ECM-related program, indicating that inferred regulon activity was not exclusive to one transcriptional program and may participate in shared aspects of remodeling. The sample-adjusted analysis reduces the influence of treating thousands of cells as independent biological replicates and instead asks whether program-associated activity recurs across patient samples. The restricted VSMC input also limits the possibility that inferred differences are driven simply by comparisons with unrelated immune or endothelial lineages. Because SCENIC does not measure transcription factor occupancy or perturbational consequences, these results should be treated as a prioritization layer rather than causal evidence. A direct functional test of the prioritized regulons would combine perturbation with program-level readouts in relevant human VSMC models. For example, *TEAD1* or *ATF3* could be reduced by siRNA/CRISPR interference or increased by controlled overexpression in primary human VSMCs exposed to plaque-relevant cues such as inflammatory cytokines, oxidized lipid, endoplasmic-reticulum stress, or altered matrix stiffness. Readouts should include contractile genes (for example, *MYH11* and *TAGLN*), ECM/remodeling genes (for example, *COL1A1*), osteogenic genes (for example, *RUNX2*), stress-response genes, proliferation/migration, and, where appropriate, calcification. ChIP-qPCR, CUT&RUN, or related chromatin assays could then test direct occupancy at predicted targets. Such experiments would distinguish whether *TEAD1* or *ATF3* merely marks a program, reinforces it, or is required for a specific remodeling response.

The independent Smart-seq2 assessment addresses an important reproducibility challenge in human single-cell studies. Droplet-based 10x Genomics and plate-based Smart-seq2 data differ in transcript capture, sensitivity, scale, and technical noise, while the two cohorts also differ in patient composition. Directly merging them could blur platform-specific structure or encourage retrospective adjustment of program definitions. We instead froze the GSE253903 signatures and confidence rules before evaluating GSE260657 and permitted uncertain cells to remain unassigned. This design produced confident assignments for 842 of 1,483 VSMC-enriched cells, leaving 641 cells (43.2%) unassigned. Importantly, unassigned is an explicit abstention state generated when either the maximum centroid similarity or the separation from the second-best centroid fails the discovery-calibrated threshold; it is not equivalent to low-quality data or absence of VSMC identity. The substantial unassigned fraction can plausibly reflect several nonexclusive factors: cross-platform differences in transcript detection and dynamic range, intermediate or mixed program expression, patient-specific states not represented by the discovery centroids, and genuine biological variation. Supplementary Figure S4 displays the confidence metrics and patient-level distribution to make this uncertainty visible. We consider this preferable to forcing every Smart-seq2 cell into one of five labels, which would overstate reproducibility. Within confidently projected cells, signature-score patterns, centroid similarities, and held-out markers supported partial conservation of the five programs. Six of seven symptomatic-versus-asymptomatic program associations were also directionally concordant across cohorts. Nevertheless, no GSE260657 module reached FDR <0.05, and centroid similarity was partly conditioned on the projection procedure. The combined evidence therefore supports partial cross-platform reproducibility at the transcriptional-program level rather than definitive identity of cell states.

Spatial transcriptomics provides an important complementary test because reproducibility of a transcriptional program does not establish where that program occurs within a plaque. Recent work by Pauli et al., including contributions from the Maegdefessel and Reilly groups, combined human carotid single-cell and Xenium spatial transcriptomics and localized inflammatory smooth-muscle and other plaque cell states within morphology-defined microenvironments (Pauli et al., 2025). The available Xenium resource uses targeted panels rather than unbiased whole-transcriptome capture, so rigorous projection of the current frozen programs would require prespecified assessment of gene-panel coverage and scoring performance. In addition, its plaque design is not identical to the matched stable-versus-ruptured regional comparison used here. We therefore did not present a *post hoc* spatial projection as equivalent to validation of the current program framework. Future work should prospectively test the frozen signatures in spatially resolved plaques together with morphology and protein imaging, particularly across fibrous-cap, calcified, inflammatory, and rupture-prone regions. The paired plaque-region analysis connects the single-cell-defined programs with a clinically relevant feature of lesion instability (Bentzon et al., 2014; Finn et al., 2010; Virmani et al., 2000; Redgrav et al., 2006). All four ruptured regions showed lower contractile scores and higher osteogenic, foam-cell-like, and proliferation scores than their matched stable regions. The paired design controls for stable patient-level characteristics and makes the directionally consistent pattern notable despite the small cohort. Reduced contractile projection is compatible with diminished representation of canonical smooth-muscle features in rupture-associated tissue, whereas higher remodeling-related projections may reflect altered matrix, lipid-associated, mineralization-associated, or proliferative activity. The simultaneous increase in several programs is also consistent with rupture regions being molecularly complex rather than dominated by one process. Because each score summarizes a predefined gene set, concordant changes across modules can reveal coordinated tissue remodeling even when no single marker is sufficient. These tissue-level patterns complement the single-cell comparison in which contractile scores were lower and several remodeling programs were higher in symptomatic plaques across both cohorts. The agreement is not exact: symptom status and local rupture are different phenotypes, and scores were calculated on different platforms and scales. Rather than implying one common mechanism, the results provide convergent association at the program level. Cell-resolved profiling of matched stable and rupture-prone regions, ideally with spatial context, will be needed to determine whether these signals arise from altered VSMC expression, redistribution of VSMC programs, changes in other cell populations, or combinations of these processes.

The potential clinical value of this framework is therefore hypothesis-generating rather than immediately diagnostic. In principle, reproducible program scores could contribute to molecular stratification of plaques beyond luminal stenosis, particularly if future prospective cohorts demonstrate that contractile loss or remodeling-program enrichment predicts imaging-defined vulnerability or recurrent ischemic events. Translation would require independent prospective validation, clinically deployable surrogates such as imaging or circulating markers, and demonstration that a program adds information beyond established clinical variables. Likewise, regulon-associated factors such as *TEAD1* or *ATF3* should not yet be viewed as therapeutic targets; targetability would require evidence that perturbation modifies plaque-relevant VSMC behavior without disrupting protective matrix or contractile functions. To provide orthogonal tissue-level RNA support for the computationally identified remodeling patterns, we performed qPCR analysis of selected VSMC program-associated genes in paired stable and ruptured plaque regions. The observed reduction of contractile markers, including *MYH11*, together with increased expression of remodeling-associated genes such as *RUNX2*, *COL1A1*, and *ATF3*, was consistent with the computationally inferred shift in expression patterns involving contractile, extracellular-matrix, osteomodulated, and stress-responsive programs. These findings provide independent RNA-level, tissue-level support for selected remodeling-associated expression patterns. They should not be interpreted as protein-level validation or proof of VSMC-specific cellular origin because qPCR was performed on whole plaque tissue. We agree that Western blotting would be valuable to determine whether selected transcript changes are reflected at the protein level, and that IHC/IF co-localization with VSMC markers such as *MYH11* or *ACTA2* would be needed to establish the spatial relationship of *RUNX2*, *ATF3*, *TEAD1*, or other candidates to smooth-muscle-associated cells *in situ*. These experiments therefore represent important orthogonal validation for future mechanistic work. Several limitations define the scope of inference. All three primary datasets were public and retrospective, and the analyses were constrained by the clinical metadata and sampling designs of the source studies. Reported race/ethnicity was incomplete and not diverse enough for a meaningful stratified analysis: public GSE253903 metadata identify nine samples as Caucasian and three as unknown, whereas race/ethnicity is not reported in the public GSE260657 sample records. Consequently, the generalizability of the program framework across ancestry and population groups remains unknown. The single-cell cohorts were cross-sectional and cannot establish the timing or direction of VSMC remodeling *in vivo*. Pseudotime depends on the selected embedding, descriptive annotations, and biologically motivated root; it represents fitted expression geometry rather than measured biological time. SCENIC infers regulon activity from co-expression and motif information and does not establish direct binding or causal regulation. The GSE260657 projection was affected by uneven patient-level cell contributions, moderate reference self-projection accuracy, and a substantial unassigned fraction. GSE120521 included only four paired patients, limiting statistical resolution; although several paired t-test results remained significant after correction, the corresponding Wilcoxon tests did not. Its bulk RNA-seq measurements reflect mixed plaque tissue, so projected scores cannot be attributed exclusively to VSMCs. Differences in sequencing platform, normalization, and transcript detection prevent direct comparison of absolute module-score magnitudes. Finally, matched chromatin, spatial, protein, and perturbational measurements were not available within the analyzed cohorts. These limitations favor interpretation in terms of descriptive VSMC transcriptional programs, partial cross-platform reproducibility, and rupture-region associations rather than causal mechanisms or clinically deployable biomarkers. The study nevertheless provides a coherent analytical framework that separates discovery, regulatory and continuous-expression characterization, independent platform assessment, and region-level plaque-instability analysis. Frozen signatures and explicit unassigned cells reduce *post hoc* optimization in the cross-platform comparison, while the paired plaque-region design provides a complementary within-patient perspective. The convergence of marker patterns, module scores, inferred regulon activity, transcriptional continua, independent projection, and targeted qPCR highlights reproducible features that can guide experimental prioritization. The same frozen framework can be applied prospectively to larger and more diverse cohorts, spatially resolved plaques, and protein or perturbational assays without redefining the discovery programs after seeing the validation data. Future work should combine spatially matched single-cell and chromatin measurements, protein localization, larger prospective cohorts with detailed plaque morphology and population metadata, and functional perturbation of prioritized regulons.

## 5 Conclusion

In conclusion, this study provides an uncertainty-aware integrative framework for characterizing vascular smooth muscle cell remodeling in human carotid atherosclerotic plaques. By separating discovery from independent cross-platform assessment and combining program-level analysis with paired plaque-region data, we identified heterogeneous VSMC transcriptional programs associated with contractile maintenance, extracellular-matrix remodeling, osteogenic and lipid-associated features, stress responses, and inflammatory signatures. Regulatory-network inference and transcriptional-continuum analyses further prioritized program-associated regulons and overlapping expression configurations. These findings do not establish causal regulatory mechanisms, spatial localization, biological lineage transitions, or a clinical biomarker; rather, they identify reproducible program-level features and explicit limits of transfer that can be tested in spatial, protein, and perturbational studies.

## Data Availability

The public transcriptomic datasets analyzed in this study are available in the NCBI Gene Expression Omnibus (GEO) repository under accession numbers GSE253903, GSE260657, and GSE120521.

## Supplementary material

SUPPLEMENTARY FIGURE S1 Quality control and broad cell annotation of human carotid plaque scRNA-seq **(A)** Sample-level fraction of cells retained after sample-aware QC and doublet exclusion in GSE253903. **(B)** Post-QC distributions of UMI counts, detected genes, mitochondrial transcript percentage, and library complexity across the 12 discovery samples; these detailed QC metrics are consolidated here from the original main-text QC panel. **(C)** scDblFinder-predicted doublet fractions among cells passing basic QC. Across all samples, 3,187 predicted doublets were excluded, leaving 46,209 cells. **(D)** Complete UMAP of retained plaque cells colored by broad cell class. **(E)** Dot plot of representative broad-lineage markers, including markers supporting mast-cell annotation. Dot size indicates the fraction of cells with detected expression and color indicates row-standardized mean expression. These panels support atlas reliability and broad annotation without implying fine-grained subtype resolution.

SUPPLEMENTARY FIGURE S2 Detailed characterization of VSMC transcriptional programs **(A)** Heatmap of completed top VSMC cluster-marker results, displaying the leading positive markers and their log2 fold-change estimates. **(B)** Dot plot of representative genes associated with the five descriptive VSMC programs: canonical contractile, ECM-associated/fibromyocyte-like, DLX-osteomodulated, stress-responsive, and interferon-associated. **(C)** Complete state-level standardized scores for the seven predefined modules. **(D)** Patient-level distribution of VSMC programs across the 12 GSE253903 samples. These proportions are descriptive and are not tissue-abundance estimates. **(E)** VSMC UMAP colored by program, including the interferon-associated subset. Program and subset terminology is used; the analysis does not establish a novel cell subtype.

SUPPLEMENTARY FIGURE S3 Detailed regulatory and transcriptional continuum analysis **(A)** Complete heatmap of mean activity for 28 motif-supported regulons across the five VSMC programs, standardized within regulon. **(B)** Sample-adjusted associations between all 28 regulons and VSMC programs; color represents the activity estimate and point size represents -log10 FDR. **(C)** Presentation of selected completed SCENIC TF-target edges for *ATF3, JUND, CREB5, TEAD1, KLF2,* and *XBP1*. Five highest-weight completed edges per TF are displayed for legibility. The network is computationally inferred and does not establish direct binding or causal regulation. **(D)** Complete Slingshot visualization of the exploratory VSMC transcriptional continua. **(E)** Heatmap of genes associated with the transcriptional continuum. **(F)** Complete predefined-module trends along pseudotime. These results describe transcriptional continua and phenotypic remodeling; they do not demonstrate lineage transition, differentiation, temporal ordering in vivo, or causality. Pathway enrichment was not included because no completed verified enrichment output was available.

SUPPLEMENTARY FIGURE S4 Detailed cross-platform projection analysis **(A)** GSE260657 UMAP views of all 1,483 selected VSMC-enriched cells, the 842 confidently projected cells, and the 641 unassigned cells. **(B)** Patient-level fractions of projected programs and unassigned cells across 15 patients. Uneven cell contributions mean these fractions should not be interpreted as tissue abundance. **(C)** Distributions of maximum reference-centroid similarity and top-versus-second-best margin, stratified by confidence class. **(D)** Pearson correlations between GSE253903 reference centroids and GSE260657 projected-group centroids. These correlations are descriptive and conditioned on projection. **(E)** Directional overlap of eight held-out reference markers with the corresponding projected group. **(F)** Complete frozen signature-score distributions across confidently projected programs. The results support partial cross-platform preservation and transcriptional similarity, not definitive validation or identity of cell states.

SUPPLEMENTARY FIGURE S5 Detailed rupture-associated VSMC program analysis **(A)** GSE120521 paired design comparing stable and visible plaque-rupture regions from four patients using frozen VSMC program scores. **(B)** Individual patient-paired scores for all seven predefined modules. **(C)** Paired contractile-program scores; all four patients showed lower scores in rupture regions. **(D)** Ruptured-minus-stable mean score differences and 95% confidence intervals for all modules. Exact paired-test and BH-adjusted values are provided in Supplementary Table S4. The analysis is exploratory and association-based (n=4 paired patients). Bulk differential expression and pathway enrichment were not included because no completed verified outputs were available.
