# Supplementary material for: Single-cell transcriptomic analysis identifies vascular smooth muscle cell transcriptional programs associated with human carotid plaque remodeling
Source: Front Mol Biosci. 2026 Sep 25;13:1950061. doi: 10.3389/fmolb.2026.1950061 (PMC13649385; doi:10.3389/fmolb.2026.1950061)
Supplement: Supplementary file 1 [file Table4.docx]

Supplementary Table S4. Primer sequences used for quantitative real-time PCR analysis

| Gene | Forward primer (5'→3') | Reverse primer (5'→3') |
| --- | --- | --- |
| MYH11 | CAGGAGCTGGAAGAGGAGAA | CCTTCTTGGCCTTCTTGTCC |
| TAGLN | AGGACTGGCAGGAGATGAGA | CTGGGCTGAGATGAGGAGGT |
| COL1A1 | GAGGGCCAAGACGAAGACAT | CAGATCACGTCATCGCACAAC |
| DCN | CAGGAGCTGGTCAAGGAGAA | GGTGATGTCGGTGTTGTTGT |
| DLX5 | CAGGAGGAGGAGGAGGAGAA | TGGGTAGGTGGTGGTGTTGT |
| RUNX2 | TGGTTACTGTCATGGCGGGTA | TCTCAGATCGTTGAACCTTGCTA |
| ATF3 | TGCCGAGATGAGCTTCCTGA | GAGGACTCCACCGTCTTCTG |
| ISG15 | GAGAGGCAGCGAACTCATCT | CTCCAGCATCTTCACCGTCA |
| GAPDH | AATCCCATCACCATCTTCCA | TGGACTCCACGACGTACTCA |
